# Squat Jump and Bilateral and Unilateral Countermovement Jump Performance in Soccer Players 6 and 9 Months After Anterior Cruciate Ligament Reconstruction

**DOI:** 10.3390/medicina62050807

**Published:** 2026-04-23

**Authors:** Nikola Andrić, Mladen Mikić, Damjan Jakšić, Slavko Molnar, Dejan Javorac, Vukadin Milankov

**Affiliations:** 1 Faculty of Sport and Physical Education, University of Novi Sad, 21000 Novi Sad, Serbia; mladen.mikic@fsfvns.edu.rs (M.M.); damjan.jaksic@fsfvns.edu.rs (D.J.); dejan.javorac@fsfvns.edu.rs (D.J.); 2 Training Expertise, 21000 Novi Sad, Serbia; 3 Faculty of Sport, University of Ljubljana, 1000 Ljubljana, Slovenia; 4 Faculty of Medicine, Department of Surgery, University of Novi Sad, 21000 Novi Sad, Serbia; vukadin.milankov@mf.uns.ac.rs; 5 Institute for Children and Youth Health Care of Vojvodina, 21000 Novi Sad, Serbia

**Keywords:** anterior cruciate ligament, soccer players, return to sport, jump performance

## Abstract

*Background and Objectives:* The assessment of neuromuscular recovery after ACL reconstruction is crucial for safe return to sport (RTS) in professional soccer players. This retrospective cross-sectional study aimed to compare squat jump (SJ), bilateral countermovement jump (CMJ), and single-leg CMJ performance in three distinct groups: players at 6 months post-ACL reconstruction, players at 9 months post-ACL reconstruction, and healthy controls. *Materials and Methods:* Seventy-two male players (24 at 6 months post-ACL, 24 at 9 months post-ACL, 24 healthy controls) performed squat jump, bilateral countermovement jump, and single-leg CMJ tests using contact platforms following a controlled warm-up protocol. *Results:* Significant group differences were observed in all jump tests. At 6 months post-ACL reconstruction, players demonstrated significantly lower squat jump (45.13 ± 6.20 cm) and bilateral countermovement jump (49.67 ± 6.80 cm) heights compared to both 9-month players (SJ: 50.03 ± 5.30 cm; CMJ: 53.79 ± 4.85 cm) and controls (SJ: 51.12 ± 4.97 cm; CMJ: 55.49 ± 5.54 cm) (*p* ≤ 0.016, η^2^ = 0.187 and η^2^ = 0.156, respectively). No significant differences between 9-month and control groups were observed for the squat jump and the bilateral countermovement jump. Regarding the unilateral countermovement jump, the injured leg showed significant performance deficits compared to controls in both the 6-month and 9-month groups (*p* = 0.001, η^2^ = 0.378). However, the non-injured leg exhibited deficits only in the 6-month group. *Conclusions:* Compared to the 6-month post-ACL reconstruction group, the 9-month group showed a marked improvement in bilateral jump performance, indicating substantial neuromuscular recovery over time. However, persistent unilateral deficits in the injured leg remained even at 9 months, underscoring the need for a routine and comprehensive jumping evaluation to identify residual neuromuscular impairments that may require targeted rehabilitation before returning to sport.

## 1. Introduction

Anterior cruciate ligament (ACL) injuries are frequent and debilitating in high-intensity sports like football requiring sprinting, jumping, and rapid direction changes [1,2]. These injuries compromise knee stability and often require surgical reconstruction for return to competition [3]. Rehabilitation targets neuromuscular deficits that elevate re-injury risk [4,5]. Despite typical RTS timelines of 6–9 months, many athletes retain impairments, underscoring the need for objective assessments within comprehensive RTS test batteries [6,7,8,9].

Lower limb power is vital for soccer-specific movements like acceleration and direction changes, making its assessment crucial in RTS evaluation [10,11,12]. While hop tests have been widely used, emerging evidence indicates vertical jump tests involve greater knee joint work and better detect power deficits during rehabilitation [13,14]. Single-leg CMJ height is significantly lower in the injured limb compared to the contralateral limb and controls, even when hop and strength assessments show symmetry. Accordingly, protocols like Aspetar recommend unilateral vertical jump testing alongside measures such as isokinetic quadriceps and hamstring torque, countermovement and drop jump height, and concentric and eccentric impulse assessments in RTS batteries [15].

The countermovement jump (CMJ) and squat jump (SJ) are commonly used to assess lower limb power in soccer players [16]. The CMJ involves a stretch-shortening cycle reflecting elastic energy use, while the SJ isolates concentric muscle power by removing pre-stretch effects [17,18]. Although the CMJ is frequently used in return-to-sport (RTS) assessments after ACL reconstruction, the SJ is less studied [15,19] despite evidence that concentric power deficits persist post-surgery and affect key soccer movements [19,20,21]. Furthermore, combining bilateral SJ and CMJ assessments with unilateral vertical jump tests may provide complementary insights into diverse neuromuscular functions essential for optimizing performance and injury prevention. Jump height remains the primary outcome metric in vertical jump testing due to its high reliability [22], strong correlation with soccer-specific performance measures such as sprinting and change-of-direction speed [23], and because related parameters derived from vertical jump testing, like power and impulse, largely reflect the same underlying construct [24]. While jump height alone does not encompass all facets of return-to-sport readiness, it provides an objective and efficient measure of critical neuromuscular recovery, making it a valuable study focus.

Limb symmetry indices (LSIs) are widely used in RTS evaluations due to their simplicity and direct interpretability. However, LSIs have important limitations, as they may overlook absolute performance deficits caused by injury-related effects on the contralateral limb, which can arise due to neural inhibition and detraining mechanisms affecting muscle strength and neuromuscular control [20]. For example, a reduced rate of force development (RFD) has been observed in the non-injured limb compared to healthy individuals during ballistic movements [25]. Therefore, including comparisons with healthy control groups provides a more comprehensive assessment of residual neuromuscular deficits following ACL reconstruction.

Several studies comparing vertical jump performance post-ACL reconstruction to matched healthy controls report inconsistent findings. Some show significant impairments in single-leg and countermovement jump heights up to nine months post-surgery [26,27,28,29], while others find no differences at later stages [30]. These conflicting results emphasize the need for further studies across multiple jump modalities and key postoperative time points to better understand neuromuscular recovery patterns [15].

While RTS decisions commonly occur between six and nine months post-surgery, the longitudinal course of jump performance recovery remains incompletely characterized. Most prior studies report outcomes at single time-points [26,27,28,30], limiting insights into persistent deficits or recovery trends. Furthermore, jump tests are typically administered in isolation at different time-points, focusing on a single assessment modality without the comprehensive comparisons of squat jump, bilateral CMJ, and unilateral CMJ performance within the same cohorts [29] or without the control group [31]. Finally, soccer imposes unique biomechanical and physiological demands due to its high-intensity, multi-directional movements and frequent jumping. Consequently, recovery patterns and functional impairments following ACL reconstruction in professional soccer players may differ from those observed in mixed-sport or recreational athlete populations. Therefore, the purpose of this study is to assess and compare the squat jump, bilateral countermovement jump, and unilateral countermovement jump performance in male professional soccer players at six and nine months following ACL reconstruction, using healthy matched controls. We hypothesize that, despite improvements over time, ACL-reconstructed players will demonstrate persistent deficits in jump height compared to controls at both time-points, with greater impairments in unilateral versus bilateral jump performance.

## 2. Materials and Methods

### 2.1. Study Design

This study employed a retrospective cross-sectional comparative design, with independent cohorts representing different post-surgical time-points: those assessed at approximately 6 months post-ACL reconstruction, those assessed at approximately 9 months post-reconstruction, and the matched healthy controls. The 6- and 9-month groups consisted of independent samples of players recruited consecutively during routine return-to-sport testing, rather than the longitudinal follow-up of the same individuals. This design allowed the comparison of neuromuscular recovery at these key post-surgical time-points while controlling for confounding variables. Testing procedures were conducted during a single visit in the morning hours in a specially designed laboratory for human sports performance evaluations in Novi Sad, Autonomous Province of Vojvodina, Serbia. The data for ACL groups were collected between February 2018 and June 2024, during regular return-to-sport assessments, while for healthy controls, the data were collected during standard pre-season measurements. Controls underwent one week of rest after the season, followed by two weeks of individual offseason training and one week of supervised team training before preseason testing, ensuring they were relatively well-trained at assessment. Standardized warm-up procedures were implemented prior to the power testing. Warm up consisted of light jogging, dynamic stretching, lower body-weight activation drills, and landing skills for 10–12 min. Participants were advised to avoid any high-intensity training sessions 72 h before the power assessment. To ensure high quality in data collection, testing procedures were supervised by an experienced rehabilitation specialist and two strength and conditioning coaches with immense experience in the elite team sports environment. A formal familiarization session was not conducted because all participants were experienced professional soccer players who regularly underwent similar jump performance assessments during their routine athletic monitoring. At testing, assessors were unaware that the data would be used for research, minimizing bias and ensuring data reliability. All jump tests were conducted in a controlled laboratory environment with an ambient temperature maintained at approximately 21 °C. The testing surface was consistent across all assessments, with participants wearing their own training shoes. The order of jump tests (squat jump, bilateral countermovement jump, single-leg countermovement jump) was fixed and standardized for all participants to minimize variability.

### 2.2. Participants

A total of seventy-two male professional soccer players competing at national and international levels, each with over ten years of playing experience, were enrolled in this cross-sectional study. Participants were divided equally into three groups: the ACL 6-month post-surgery group (*n* = 24), the ACL 9-month post-surgery group (*n* = 24), and the healthy control group (*n* = 24). The ACL groups consisted of athletes consecutively recruited from patients attending rehabilitation at the clinic “Trenazna ekspertiza-Novi Sad”. Participants in the 6-month and 9-month post-ACL reconstruction groups were distinct individuals who underwent testing only once at their respective time-point. No participant was assessed at both time-points. Although some participants underwent testing at both 6 and 9 months post-ACL reconstruction, this subgroup was relatively small and insufficient for a robust longitudinal analysis. To maximize statistical power and representativeness, we therefore opted for a cross-sectional design with independent cohorts evaluated at either 6 or 9 months. This approach allowed for more comprehensive group comparisons while reflecting the clinical realities of rehabilitation scheduling within our clinic. Frequency matching was conducted, with controls recruited from the same competitive league and training environments as the ACL participants to ensure the comparability of athletic background and physical conditioning.

For inclusion in the ACL groups, participants had to meet the following criteria: primary unilateral ACL reconstruction performed using a bone–patellar tendon–bone (BPTB) graft; clearance for testing from an orthopedic surgeon and a sports medicine specialist, with no knee pain or swelling at the time of assessment; and the completion of a standardized rehabilitation program designed for ACL recovery. The control group participants were included if they had no history of knee or lower limb injury in the six months preceding testing; were free from illness for at least 14 days prior to testing; and were actively engaged in professional soccer training and strength and conditioning programs. Exclusion criteria for all groups included current musculoskeletal injuries, neuromuscular impairments unrelated to the ACL injury, or inability to perform jump tests safely. All patients followed a standardized and progressive rehabilitation program [15]. Rehabilitation progressed through evidence-based phases targeting pain and swelling control, the restoration of range of motion, strength gains—including both open and closed kinetic chain exercises—and neuromuscular control. Eccentric training, motor control exercises, and plyometric and agility drills were incorporated in later stages to enhance muscle function, balance, and sport-specific performance. Objective progression criteria, including functional testing and patient symptoms, guided advancement through recovery phases and return-to-sport clearance. Although minor individual variations in rehabilitation adherence and training load existed, all athletes had completed rehabilitation phases sufficient to commence return-to-sport testing. Participant characteristics are summarized in Table 1.

Body mass did not differ significantly between groups, therefore jump height analyses were performed on absolute values. All participants were informed about the testing procedures, and that the collected data could be used for scientific research. Written informed consent was obtained prior to participation. The study protocol was approved by the Ethics Committee of the Faculty of Sport and Physical Education, University of Novi Sad, Serbia (protocol number: 52-02-11/2026-1). This study complied with the Declaration of Helsinki and institutional guidelines for research with human subjects.

### 2.3. Testing Procedures

The squat jump (SJ) was conducted using contact platforms (Just Jump, Probotics, Huntsville, AL, USA). This system has demonstrated excellent test–retest reliability in measuring vertical jump performance in team sport athletes, with minimal test–retest bias (Mean bias (95% CI) = 0.74 cm (0.08–1.42 cm)) [32]. To eliminate the potential stretch-shortening cycle mechanism, participants started the test in the standing position, and the on tester’s command, they slowly went downward in the semi-squat position, with around 90 degrees of knee and hip flexion (visual feedback), held that position for two seconds, and then maximally accelerated their body and jumped up into the air using explosive concentric contractions of lower limbs and landed in the knee-extended position. Participants performed 3 trials, and the best jump performance (jump height), measured in cm, was reported and used for further analysis. The rest pause between each trial was 45 s.

The countermovement jump (CMJ) was conducted according to the Bosco protocol, using contact platforms (Just Jump, Probotics, Huntsville, AL, USA, USA). Participants started the test in the upright position with their hands on their hips throughout the entire test. From the starting position, participants swiftly went downward to a self-selected semi-squat position, then maximally accelerated their body and jumped up into the air using the powerful action of lower limbs, and landed in the knee-extended position. Participants were advised to minimize the time period between the eccentric and concentric phases. Participants performed 3 trials, and the best jump performance (jump height), measured in cm, was reported and used for further statistical analysis. The rest pause between each trial was 45 s.

The single-leg countermovement jump (SL CMJ) was performed using contact platforms (Just Jump, Probotics, Huntsville, AL, USA). Participants were instructed to always keep their hands on the hips during the test and not to swing the opposite leg during the jump. The test started in the upright position with the right/left leg on the ground and with the left/right leg in the air and flexed at 90 degrees in the knee joint. Participants were advised to swiftly move downward into the self-selected semi-squat position and then jump up maximally in the air, landing on the jumping leg with the knee in the extended position. Participants performed 3 trials, and the best performance measure (jump height), expressed in cm, was documented and used for further statistical analysis. The rest pause between each trial was 45 s.

All jump tests have been widely validated in both research and clinical settings as reliable and sensitive measures of lower limb power and neuromuscular function [13,18]. The injured limb was analyzed separately from the non-injured limb for the unilateral jump tests. For healthy controls, the dominant limb—determined by participants’ preferred kicking leg—was compared with the injured limb of the ACL groups. The contralateral limb in ACL participants and the non-dominant limb in controls were also compared.

### 2.4. Statistical Analysis

All raw and processed data used for analysis in this study are available in the Supplementary Material (excel file). Data were analyzed using SPSS version 29 (IBM Corp., Armonk, NY, USA). Descriptive statistics are presented as means ± standard deviations (SDs) and 95% confidence intervals (CIs). Normality of data distribution and homogeneity of variances were assessed using the Shapiro–Wilk and Levene tests, respectively, confirming that assumptions for parametric analysis were met. To compare jump performance (squat jump and countermovement jump heights) among the three groups (ACL 6-month, ACL 9-month, and healthy controls), a one-way analysis of variance (ANOVA) was performed. When significant group effects were detected, post hoc pairwise comparisons were conducted using the Tukey HSD test to identify specific between-group differences. Effect sizes for these pairwise comparisons were calculated using eta-squared (η^2^) to quantify the magnitude of differences, interpreted as small (0.01), medium (0.06), and large (0.14) effects. Additionally, a post hoc power analysis was conducted using the software G*Power (version 3.1, Heinrich-Heine-Universität Düsseldorf, Düsseldorf, Germany) to estimate the achieved statistical power (1 − β) for detecting differences between groups. The analysis used observed effect sizes (η^2^), group sample sizes (*n* = 24 per group), and an alpha level of 0.05 for two-tailed tests. Significance was accepted at *p* < 0.05 for all tests.

## 3. Results

**Figure 1:**
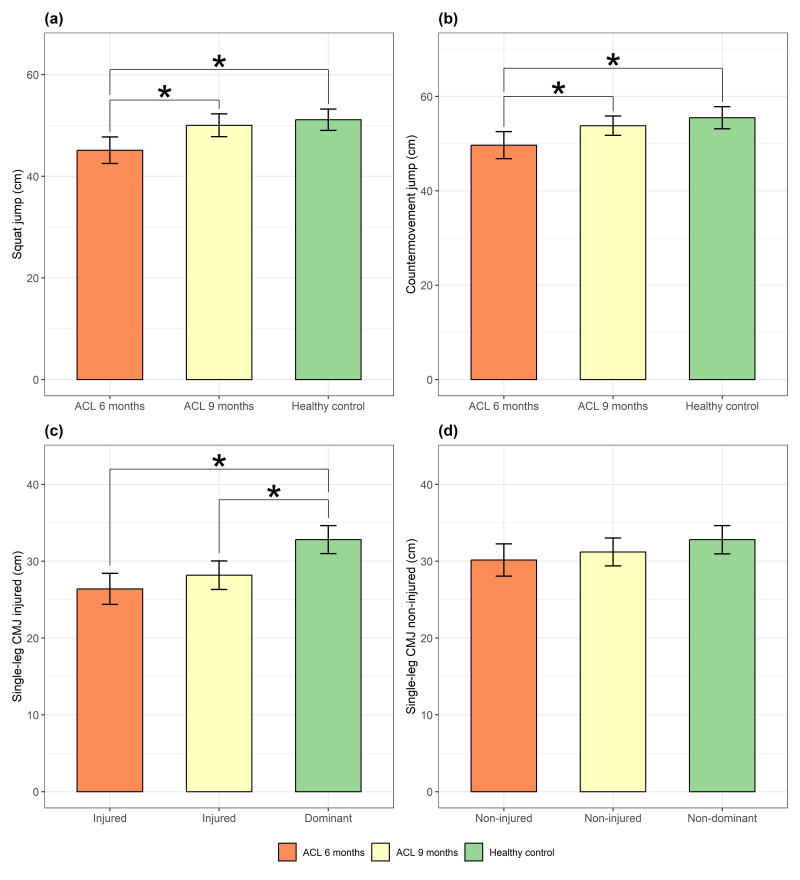
The differences in bilateral and unilateral vertical jump performance across groups: (**a**) squat jump; (**b**) countermovement jump; (**c**) single-leg CMJ (injured limb); and (**d**) single-leg CMJ (non-injured limb). Values are presented as mean ± 95% CI. ∗—significantly different (*p* < 0.05).

The one-way ANOVA showed a significant effect of group on the SJ (F = 8.03, *p* = 0.001, η^2^ = 0.187), CMJ (F = 6.42, *p* = 0.003, η^2^ = 0.156), and injured SL CMJ (F = 12.935, *p* = 0.001, η^2^ = 0.271). For the non-injured SL CMJ, the ANOVA did not reach statistical significance (F = 2.06, *p* = 0.135, η^2^ = 0.059) (Table 2). Post hoc comparisons using the Tukey HSD test revealed a significant difference for the SJ between the ACL 6-month group and the ACL 9-month group (*p* = 0.003, η^2^ = 0.190), as well as between the ACL 6-month group and the control group (*p* = 0.001, η^2^ = 0.261), with no significant differences between the ACL 9-month group and the control group (*p* > 0.05) (Figure 1a,b).

Similarly, for the countermovement jump, post hoc tests revealed a significant difference between the ACL 6-month group and the ACL 9-month group (*p* = 0.016, η^2^ = 0.132) and between the ACL 6-month group and the control group (*p* = 0.001, η^2^ = 0.232) (Figure 1). For the single-leg countermovement jump of the injured leg, post hoc tests revealed a significant difference between the ACL 6-month group and the control group (*p* = 0.001, η^2^ = 0.378) and between the ACL 9-month group and the control group (*p* = 0.001, η^2^ = 0.366), (Figure 1c,d).

To evaluate the robustness of these findings, a post hoc power analysis was conducted using the software G*Power (version 3.1). Based on the observed effect sizes (η^2^ ranging from 0.093 to 0.378), the group sample sizes (*n* = 24 per group), and an alpha level of 0.05, the estimated statistical power to detect differences varied between 71% and 97%. Specifically, power was estimated at 88% and 97% for the SJ comparisons and 71% and 93% for the CMJ comparisons. Importantly, for the single-leg countermovement jump (SL CMJ) of the injured leg, large effect sizes were observed (η^2^ = 0.378 for ACL 6-month vs. controls and η^2^ = 0.366 for ACL 9-month vs. controls), resulting in high statistical power exceeding 97%. These results indicate that this study was sufficiently powered to detect moderate-to-large differences in the SJ, bilateral CMJ, and unilateral SL CMJ performance between the groups.

## 4. Discussion

This study cross-sectionally compared the squat jump (SJ), bilateral countermovement jump (CMJ), and single-leg countermovement jump (SL CMJ) performance in professional soccer players assessed at either 6 or 9 months following anterior cruciate ligament reconstruction (ACL) with matched healthy controls. The 6-month post-ACL group exhibited significantly reduced SJ and bilateral CMJ heights compared to both the 9-month post-ACL group and healthy controls; no differences were observed between the 9-month group and controls. Additionally, the SL CMJ performance of the injured limb showed significant deficits relative to controls for both 6- and 9-month post-ACL groups. The non-injured limb showed no significant differences between groups. These results indicate that, while bilateral lower limb concentric power and slow stretch-shortening cycle function appear to improve between these cross-sectional time-points, neuromuscular deficits in the unilateral function of the injured limb persist beyond 9 months. While these findings represent group-level differences, they may tentatively suggest longitudinal recovery trajectories; however, definitive conclusions cannot be drawn from this cross-sectional design.

Several longitudinal studies have reported changes in vertical jump performance during the late stages of ACL rehabilitation. Edwards et al. [31] observed modest improvements in CMJ height in 91 non-elite team sport athletes at 9 months compared to 6 months post-ACL surgery. Similarly, Read et al. [29] assessed 370 professional soccer players grouped by time since ACL reconstruction (<6 months, 6–9 months, and >9 months) and found that, although CMJ height progressively improved, it remained significantly lower than that of matched healthy controls even in the later recovery stages (30.7 ± 6.4 cm vs. 34.5 ± 6.0 cm; *p* < 0.05). Dutaillis et al. [25] followed recreational athletes for 12 months post-ACL surgery and reported significant improvements in CMJ and SL CMJ performance over time; however, deficits and asymmetries in the single-leg jump height of the operated limb persisted throughout rehabilitation. Legnani et al. [26] found that limb symmetry indices for SL CMJ height were unsatisfactory in most patients at 6 months post-ACL, with most improving and meeting return-to-sport criteria by 12 months. Collectively, the evidence indicates that bilateral jump performance largely recovers between 6 and 9 months post-ACL, whereas unilateral deficits persist beyond this timeframe, consistent with our results. While comparisons with longitudinal studies provide context, the cross-sectional design of the present study limits the ability to track individual recovery patterns. Therefore, the results should be interpreted as reflecting differences between groups at discrete time points rather than definitive recovery trajectories.

Several other investigations have assessed various vertical jump metrics in ACL-injured populations relative to healthy controls, with conflicting results. Kotsifaki et al. [13,27] evaluated male athletes at around 9 months post-ACL surgery and reported that involved limbs exhibited significantly lower single-leg vertical jump height and reactive strength index compared to both the uninvolved limb and healthy controls. Maestroni et al. [33] evaluated professional soccer players at return to sport, approximately 9 months post-ACL reconstruction. They reported that, while bilateral countermovement jump height recovered to preinjury levels, the single-leg countermovement jump height of the injured limb remained about 12% lower than the preinjury height and was significantly reduced compared to healthy controls. Similarly, O’Malley et al. [28] compared 118 male athletes at least 6 months post-ACL reconstruction with 44 healthy controls. They reported that ACL athletes exhibited significantly lower single-leg countermovement jump (SL CMJ) height and limb symmetry indices than controls, with moderate (d > 0.4) and large effect sizes (d > 1.1), respectively. Conversely, Cabarkapa et al. [30] found no significant differences in CMJ force–time metrics or asymmetries between ACL-reconstructed semi-professional soccer players and healthy controls 11–13 months post-surgery, suggesting that the countermovement jump alone may lack sensitivity to detect neuromuscular deficits late in the rehabilitation process. Taken together, these findings align with our results and highlight the necessity of comprehensive jump assessments to guide targeted rehabilitation and safe return-to-sport decisions. The similar recovery patterns observed between squat jump (SJ) and bilateral countermovement jump (CMJ) performance likely reflect the comparable biomechanical and neuromuscular demands of these tasks, both involving bilateral concentric power production under symmetrical loading conditions [18]. The higher jump heights observed in the CMJ compared to the SJ have been attributed to the greater hip joint work during the initial 30% of muscle shortening in the CMJ, resulting from increased muscle activity and force generation prior to the propulsive phase. In contrast, force development in SJ occurs progressively throughout the propulsive phase [17]. Although the SJ and CMJ assess distinct movement qualities, a shared variance of 47.6% between their jump heights indicates a substantial overlap in the neuromuscular factors they measure [34]. This substantial overlap likely contributes to why various training regimens, including weightlifting and plyometric exercises, yield similar improvements in both SJ and CMJ performance [35,36].

Single-leg countermovement jump (SL CMJ) for injured leg showed persistent deficits in jumping performance in the 9-month group, suggesting that unilateral function may recover more slowly. This divergence is likely due to the greater functional demands and increased neuromuscular complexity inherent in unilateral tasks. Executing a single-leg jump requires a substantially higher relative force output—estimated to be approximately 1.62 times that of a bilateral CMJ—to vertically displace body mass [37], highlighting the significant contribution of lower body strength to SL CMJ performance outcomes [38]. While this highlights the need for targeted unilateral eccentric-to-concentric training in rehabilitation, the inclusion of such specific exercises was not detailed in our study and warrants further investigation. Furthermore, previous studies report that absolute strength is a better determinant of single-leg jump height than bilateral CMJ height [39,40]. It is plausible that strength levels achieved by the time of return to sport are sufficient to restore CMJ performance to levels comparable to those of healthy controls, but they remain inadequate to normalize single-leg jump height.

Several unique biomechanical and neuromuscular challenges arise during single-leg compared to double-leg jumps. While hip extensor strength is a key factor in general jump performance [41], single-leg jumps place greater emphasis on the hip abductors, external rotators, and lateral trunk flexors, which contribute to frontal plane stability and dynamic knee valgus control [42]. Additionally, the neural drive during single-leg jumps has been shown to be significantly higher than that during double-leg jumps, reflecting greater neuromuscular demand and motor control requirements [43]. The increased demands on strength within these smaller muscle groups, as well as on stability and motor control necessary for single-leg tasks, may be inadequately targeted during standard rehabilitation protocols that typically focus on bilateral strength training modalities [44]. Consequently, this insufficient emphasis likely contributes to the prolonged and incomplete recovery observed in single-leg jump performance following ACL reconstruction.

This study has several limitations that warrant consideration. First, the cross-sectional design involving independent cohorts at 6 and 9 months post-ACL reconstruction precludes tracking individual recovery trajectories. Second, this study centered predominantly on jump height as the primary outcome measure. Although prior research demonstrated that jump height is a reliable and valid indicator of lower limb power and neuromuscular function [22,24], incorporating additional biomechanical parameters—such as power output, rate of force development, and kinetic or kinematic variables—would offer a more comprehensive characterization of functional deficits. Future research should aim to integrate these additional metrics. Third, the potential for contextual bias exists due to differences in testing conditions; ACL participants were assessed during return-to-sport evaluations conducted over several years, while healthy controls were evaluated during standard pre-season assessments. This discrepancy may reflect variations in training status, fatigue level, and seasonal conditioning that could independently affect jump performance. Caution is therefore warranted when interpreting between-group comparisons. Fourth, limiting the cohort to bone–patellar tendon–bone (BPTB) graft recipients improves internal validity but restricts the generalizability of the findings to players undergoing hamstring or quadricep tendon autograft procedures. Finally, our study prioritized absolute comparisons with healthy controls to effectively capture neuromuscular deficits. We acknowledge that omitting Limb Symmetry Index (LSI) calculations may limit direct comparison with established benchmarks; this limitation should be considered when interpreting our findings.

This study’s findings underscore the complexity of neuromuscular recovery following ACL reconstruction, highlighting that while bilateral power may normalize by 9 months, unilateral impairments persist and could elevate reinjury risk. Recognizing these nuanced recovery patterns is important for clinicians designing targeted rehabilitation interventions. Future research should explore tailored training approaches addressing unilateral deficits and investigate longer-term functional outcomes to optimize return-to-sport safety.

## 5. Conclusions

Significant differences in SJ and CMJ height were observed between the groups evaluated at 6 and 9 months post-ACLR, with the 9-month group exhibiting jump heights comparable to those of healthy controls. However, persistent deficits in SL CMJ remain in the injured limb, suggesting that rehabilitation programs should specifically target unilateral strength and neuromuscular control to address these lingering impairments. Importantly, due to the cross-sectional design using independent cohorts, these findings reflect group differences at specific time points rather than individual recovery trajectories. Therefore, conclusions regarding individual neuromuscular recovery timelines remain tentative and warrant confirmation through longitudinal studies. Incorporating a diverse array of vertical jump assessments and encompassing both bilateral and unilateral tests enhance the detection of subtle neuromuscular impairments and support individualized, evidence-based rehabilitation and return-to-sport decision making.

## Figures and Tables

**Table 1:** Patient information and the results of one-way ANOVA for body weight.

	ACL 6 Months (*n* = 24)	ACL 9 Months (*n* = 24)	Healthy Control	F (*p*)
Age (years)	23.0 ± 5.0	21.8 ± 2.8	22.9 ± 4.0	
Body weight (kg)	80.3 ± 6.2	77.3 ± 8.6	75.6 ± 6.0	2.75 (0.071)
Graft type	BPTB	BPTB	N/A	
post-surgery (days)	197.2 ± 18.5	267.5 ± 28.9	N/A	

BPTB—bone–patellar tendon–bone graft; N/A- not applicable.

**Table 2:** The results of one-way ANOVA for squat jump, countermovement and single-leg countermovement jump performance. Values are presented as means and standard deviations.

Jump	ACL 6 Months		ACL 9 Months		Healthy Control		F	*p*
	Mean (SD)	CI	Mean (SD)	CI	Mean (SD)	CI		
SJ (cm)	45.13 (6.20)	[42.51, 47.75]	50.03 (5.30)	[47.80, 52.27]	51.12 (4.97)	[49.02, 53.22]	8.032	0.001
CMJ (cm)	49.67 (6.80)	[46.80, 52.54]	53.79 (4.85)	[51.75, 55.84]	55.49 (5.54)	[53.15, 57.82]	6.421	0.003
SL CMJ injured (cm)	26.40 (4.79)	[24.38, 28.43]	28.19 (4.38)	[26.33, 30.04]	32.81 (5.20)	[30.99, 34.64]	12.935	0.001
SL CMJ non-injured (cm)	30.16 (4.99)	[28.06, 32.26]	31.21 (4.29)	[29.40, 33.02]	32.81 (4.40)	[30.96, 34.64]	2.058	0.135

SJ- Squat jump; CMJ- Countermovement jump; SL CMJ- Single-leg countermovement jump.

## Data Availability

The data are available upon reasonable request.

## Supplementary Materials

The following supporting information can be downloaded at: https://www.mdpi.com/article/10.3390/medicina62050807/s1.
